# Spicy and Aromatic Plants

**DOI:** 10.3390/plants12040848

**Published:** 2023-02-14

**Authors:** Romina A. Marc, Crina C. Mureșan, Andruța E. Mureșan

**Affiliations:** Food Engineering Department, Faculty of Food Science and Technology, University of Agricultural Science and Veterinary Medicine Cluj-Napoca, 3-5 Calea Mănăștur Street, 400372 Cluj-Napoca, Romania

## 1. Introduction

The purpose of this Special Issue entitled “Spicy and Aromatic Plants” is to expand our knowledge about edible plants, which have been used for thousands of years, by all the peoples of the world, in every household. They give special flavors to culinary preparations and have various curative properties. This Special Issue contains six scientific articles (five original research articles and one original review), which contribute the knowledge of edible plants used in fields, such as the food, pharmaceutical, cosmetic, and agricultural industries. This editorial aims to summarize the valuable work that is published in this Special Issue, and to increase the visibility and citations of these studies.

## 2. Overview of the Special Issue

Puscaș et al. (2022) [1] published a research paper entitled “Cytotoxicity Evaluation and Antioxidant Activity of a Novel Drink Based on Roasted Avocado Seed Powder”. The paper describes the creation and utilization of avocado seed in a hot drink, similar to already-existing coffee alternatives, obtained by infusing roasted and ground avocado seeds. Different time and temperature protocols for roasting avocado seeds were evaluated, along with drying, as were changes in flavor and color. It was proposed that the powder of roasted avocado seeds be valorized in a hot drink (at 180 °C for 25 min) by making an infusion of 7% powder and hot water. Raw and conditioned avocado seeds, as well as the resulting drinks, were analyzed. Seeds have large amounts of carbohydrates, including dietary fiber. The percentage of proteins determined was 4–5%, with the difference being dependent on the process applied: drying or roasting. Flax in the raw seed was determined to have the highest content of polyphenols (772.90 mg GAE/100 g). The amount of polyphenols in the drink was much lower (17.55 mg GAE/100 g). The antioxidant capacity of the drink was high. The acidity and total carotenoid compound increased significantly during conditioning. The antioxidant capacity of the drink was high (90.27 RSA%), which could be due to the high content of total carotenoid compounds detected in the roasted seed (6534.48 µg/100 g) or flavonoids. The 7% roasted avocado seed powder drink was shown to have high antiproliferative activity in Hs27 and DLD-1 cell lines. These values are higher than the previously reported values for coffee and coffee substitutes in other studies.

Saqib et al. (2022) [2] published a research paper entitled “Antidiarrheal and Cardio-Depressant Effects of *Himalaiella heteromalla* (D.Don) Raab-Straube: In Vitro, In Vivo, and In Silico Studies”. *Himalaiella heteromalla* (D.Don) Raab-Straube is commonly known as Batula. Its use dates back a long time as an adjuvant in the treatment of various diseases. In this work, the crude extract of *H. heteromalla* and its fractions were investigated for their gastrointestinal, bronchodilator, cardiovascular, and anti-inflammatory activities. *H. heteromalla* crude extract (Hh.Cr) dose-dependently relaxed spontaneous contractions and K^+^ (80 mM)-induced contraction in the jejunum tissue. The relaxation of K^+^ (80 mM) indicates the presence of a Ca^++^ channel blocking (CCB) effect, which was further confirmed by constructing calcium response curves (CRCs) as they caused a rightward parallel shift in CRCs in a manner comparable to verapamil; so, the spasmolytic effect of Hh.Cr was due to its CCB activity. The application of Hh.Cr in CCh (1 µM)- and K^+^ (80 mM)-induced contraction in tracheal preparation resulted in complete relaxation, showing its bronchodilator effect, which was mediated through Ca^++^ channels and cholinergic antagonist activity. The application of Hh.Cr in aortic preparations resulted in vasorelaxant activity through angiotensin and α-adrenergic receptor blockage. It also had a cardio-suppressant effect with negative chronotropic and inotropic responses in paired atrium preparation. Similar effects were observed in in vivo models, i.e., decreased propulsive movement, wet feces, and the inhibition of edema formation. *Himalaiella heteromalla* had a more spasmolytic effect in the ethyl acetate fraction and caused complete relaxation of the isolated jejunum, trachea, aorta, and paired atria; this was supported by in silico studies.

Ciocarlan et al. (2021) [3] published a research paper entitled “Chemical Composition and Assessment of Antimicrobial Activity of Lavender Essential Oil and Some By-Products”. The purpose of the present study was to analyze the chemical composition of lavender *(Lavanda angustifolia* L.) essential oil and some by-products derived from its production (residual water and residual herbs), as well as to assess their “in vitro” antimicrobial activity. Lavender samples from seven industrial producers from Moldova were analyzed and 41 essential oil compounds were identified. The method used was gas chromatography–mass spectrometry. Significant antimicrobial activity was identified for *Bacillus subtilis, Pseudomonas fluorescens*, *Xanthomonas campestris*, *Erwinia carotovora*, *Erwinia amylovora*, and *Candida utilis*. Antimicrobial activity of lavender plant material, wastewater, and ethanol extracts from solid waste residue was observed for *Aspergillus niger*, *Alternaria alternata*, *Penicillium chrysogenum*, *Bacillus* sp., and *Pseudomonas aeruginosa*. Antimicrobial activity differed depending on the concentrations.

Mushtaq et al. (2021) [4] published a research paper entitled “Biomolecular Evaluation of *Lavandula stoechas* L. for Nootropic Activity”. The aim of this study was to identify the biomolecules of lavender that are responsible for improving memory. An aqueous extract of *L. stoechas* was made, which was first purified, and then, analyzed in vitro for anticholinesterase activity. An active fraction of *L. stoechas* (AfL.s) was subjected to biomolecule analysis using the GS-MS method. Two main compounds were identified: α-tocopherol and phenethylamine. These compounds were administered to mice at different doses for two days. The mice were sacrificed and their brains were analyzed for a biochemical assay. α-Tocopherol reduces free radical oxidative stress in the brains of mice, while phenethylamine increases the levels of acetylcholine in the hippocampus. Thus, it was concluded that α-tocopherol and phenethylamine (a primary amine) present in *L. stoechas* improved the memories of the studied animals and can be used as a memory enhancer.

Sriwichai et al. (2021) [5] published a research paper entitled “Aromatic Profile Variation of Essential Oil from Dried Makwhaen Fruit and Related Species”. This work was carried out to evaluate the relationships between the genotype, phenotype, and chemical profiles of essential oil obtained from Zanthoxylum spp. A morphological comparison was carried out for three specimens of Makhwaen (MK) with other Zanthoxylum spices: Huajiao (HJ) and Makwoung (MKO). The extracted essential oils were analyzed from a chemical and physical point of view. MKO and MK showed similar volatile profiles of fruity, woody, and citrus aromas, while HJ was distinctive with a citrus-floral aroma.

Marc et al. (2022) [6] published a research paper entitled “Spicy and Aromatic Plants for Meat and Meat Analogues Applications”. This review paper aimed to present updated information on the antioxidant and antimicrobial properties of the most common herbs and spices (parsley, dill, basil, oregano, sage, coriander, rosemary, marjoram, tarragon, bay, thyme, and mint) used in the meat and meat analogue industries.

## 3. Conclusions

The growing interest of consumers, processors, and producers in spicy and aromatic plants makes this Special Issue highly interesting. These plants are appealing due to their natural aromas, antioxidants, and antimicrobial substances, as well as their demonstrated bioactive compounds. The use of these plants is recommended due to their ability to improve flavor, and for their health benefits to consumers. We are pleased that our published papers have different numbers of citations, which demonstrates their quality and the interest of readers and scientists.
